# An analysis of the spread of electric field within the cochlea for different devices including custom-made electrodes for subtotal cochleoectomy

**DOI:** 10.1371/journal.pone.0287216

**Published:** 2023-09-08

**Authors:** Luise Wagner, Stefan K. Plontke, Torsten Rahne

**Affiliations:** Department of Otorhinolaryngology and Halle Hearing and Implant Center, Martin-Luther-University Halle-Wittenberg, Halle (Saale), Germany; Justus Liebig University Giessen, GERMANY

## Abstract

**Objective:**

Cochlear implants (CIs) can restore hearing not only in patients with profound hearing loss and deafness, but also in patients following tumour removal of intra-cochlear schwannomas. In such cases, design and placement differ from conventional electrode insertion, in which the cochlea remains filled with fluid. Despite these technical and surgical differences, previous studies have tended to show positive results in speech perception in tumour patients. The purpose of this study is to retrospectively evaluate the ability to predict speech recognition outcomes using individual electric field spreads and to investigate worldwide unique tumour cases.

**Study design:**

In a retrospective analysis in a university tertiary center electric field spreads were compared between two groups of electrode designs implanted between 2009 and 2020 i.e., between lateral wall electrodes and custom-made perimodiolar electrode carriers from the same company. The voltage gradients were analysed and grouped with speech recognition results.

**Results:**

Differences in electrical field spreads were found between lateral wall electrodes and the custom-made perimodiolar electrodes, whereas a significant influence of electric fields on scores in speech recognition cannot be demonstrated.

**Conclusion:**

Prediction of speech recognition outcome based on electric field propagation results seems not feasible. Significant differences in field spread between electrode arrays can be clearly demonstrated. This observation and its relevance to hearing treatment and speech recognition should therefore be further investigated in upcoming studies.

## Introduction

### Cochlear implants and their limitations

Cochlear implants (CIs) can restore hearing not only in patients with severe to profound hearing loss and deafness, but also in patients after tumour removal of intra-cochlear schwannomas, that are benign. These tumours originate in the peripheral branches of the eighth cranial nerve and often require surgical excision, resulting in partial or subtotal cochlear removal [1]. Interestingly, affected patients who were treated with CIs can potentially demonstrate comparable outcome performance as single-sided deafened CI users [1, 2] and sometimes even better.

To evoke a sound perception in CI users, cochlear nerve fibres are stimulated by electrical current pulses along the electrode array. Electrode contacts close to the modiolus as provided by so-called perimodiolar electrode arrays achieve a more precise stimulation of a smaller amount of nerve fibers compared to straight electrode carriers that deliver the pulses with greater distance to the modiolus [REF]. Apical electrodes of longer electrode carriers achieve more apical nerve fibers and therefore are supposed to achieve a better low-frequency hearing than shorter electrodes [REF]. The electrical impedance of the electrode-tissue interface including the cochlear fluids determines the absolute values of current flow within the cochlea. Thus, even in cochleae with regular anatomy the current spread can vary between a focussed stimulation of a certain array and an excitation of nerve fibres that can span about one third of the electrode array or even more [3, 4]. Unfortunately, this undifferentiated spread leads to channel interaction, restricts spectral resolution and all together leads to inaccurate auditory representation—still a limiting factor in current CI technology [5]. Therefore, the enhancement of speech and music perception based on improved spatial resolution in CIs is widely discussed [6, 7].

### Theories of influences on the intra-cochlear current flow

The CI induced current spread and flow within the cochlea was modelled in several studies (see review [8]). This is based on different influencing factors, e.g., electrical resistivity of the bone and tissue [9, 10], electrical conductivity of the modiolus encapsulation [9], and changes in electrode-electrolyte interface by charge injection [8, 11]. Other like Choi et al. [12] used finite elements to model electrical impedances across the electrode array based on the assumption that the distance between electrode array and modiolus stays constant along its progression. However, the validity of this assumption has been questioned in other studies [13, 14]. Nevertheless, using these insights, Mens [15] was able to show strong gradient related field spreads, where the apical to basal voltage propagation shows an increase in amplitude of about three times. These results strongly emphasize the necessity of further investigations into such mechanisms and effects.

In addition to electrochemical and physiological factors one can hypothesize that the actual design of the CI electrode array influences the spread of electric field. During the last decades CI manufacturers developed and offered a variety of different electrode designs, e.g., long lateral wall arrays with the aim to cover the complete cochlear length and nerve fibres (MED-EL, Austria), shorter perimodiolar electrode arrays to decrease distance between carrier and nerve fibres (Cochlear, Australia), and others. There are study results comparing the difference in word recognition between perimodiolar and lateral wall electrode designs finally base on the different electrical conditions within the cochlea showing facilitated word recognition in perimodiolar arrays [7].

To measure electrical impedances and voltage spreads in vivo CI manufacturers offer system integrated telemetry functions, that are commonly used in daily clinical routine to monitor correct implant functioning. One particular telemetry feature is the measurement of the intra-cochlear electric field or intra-cochlear current spread (ICCS) [16], labelled different for different manufacturers like electrical field imaging (EFI) or transimpedance matrices (TIM) [17] or voltage matrices. Although different labelled the ICCS results seem to be similar for all manufacturers [18]. All these tools measure the impedance and voltage gradient along the electrode array for the respective CI system. These values give no direct information about the activation of the nerve fibres because it is a purely physical measure. Nevertheless they are related with the neural spread of excitation [19]. In MED-EL devices this measure is included in each routine impedance measure.

### Specialties after subtotal cochleoectomy

All above mentioned findings are based on the fact that the underlying anatomy of the cochlea is intact and its compartments remain fluid-filled. In contrast, in tumour patients with partial or subtotal cochleoectomy, the fluid-filled compartments are degraded and thus their contribution to the electric field spread is impaired. One would expect a poor outcome but several studies have shown the opposite [1, 2, 20]. These patients could particularly benefit from precise control of the electric fields and thus from differential recruitment of the nerve fibres. For those patients, a perimodiolar electrode array was developed by MED-EL (Innsbruck, Austria) as a custom made device (CMD) based on the template of their common FORM19 electrode array [21].

Interestingly, significant peculiarities in the electrical field spread were shown across the different patient groups. For instance, Wagner et al. [2] observed smaller electric fields in Nucleus CI patients after partial or subtotal cochleoectomy for removal of intra-cochlear schwannoma compared to patients who underwent standard cochlear implantation through a round window approach. Noteworthy, patients who underwent cochleoectomy at cochlear implantation were able to demonstrate comparable or even better outcome in speech perception compared to the group with “normal” CI insertion [1].

For patients after standard implantation approaches Da Silva et al. [22] investigated not just the technical field but the excited fibres which give insight in the real transmission and found that more precisely differentiated areas of recruited nerve fibres correlate with better outcomes in speech perception using spread of excitation measurements. Joly et al. [16] found a correlation between speech perception and the current spread. However, the exact effects of stimulation field spreads and spread of excitation areas are far from being understood. Therefore, it is of deeper interest of this study to further investigate the relationship of technical electrical field spreads with speech perception results, not regarding the excitation and all other known factors for speech perception results, like e.g. duration of deafness, ethiology, age at implantation [23, 24]. Of course all these factors play a significant role for speech perception outcome and their influence on word recognition is discussed a lot e.g. in Holden et al. [25] but as Joly et al. [16] said ICCS alone can explain 30% of speech intelligibility.

### The aim of the study

To have a robust fundament for analysis two different patient groups were used, one group with commonly implanted CIs by standard surgical approaches using standard electrode arrays as well as one group after subtotal cochleoectomy using special CMD electrode arrays (both provided by MED-EL). This combination offers an innovative and rare approach in research regarding this topic as these special arrays in patients are one of a kind worldwide. Noteworthy, at the time of implantation only MED-EL implant technology allowed postoperative MRI diagnostics, what was essential for the follow-up therapy of tumour patients. Because these intra-cochlear schwannomas are a very rare disease the sample size is quite small. In summary, we expect to find differences in provided field spreads among the two investigated arrays based on their design and patient related anatomy as well as deeper insights into the relationship to speech performance outcomes.

## Methods

### Study design and patients

In a retrospective exploratory case-control study CI users with MED-EL devices were investigated. All patients were implanted between 02/2009 and 11/2020 at a tertiary university referral centre. All datasets were scanned for validity and included if a valid IFT measurement as well as a word recognition score test (Freiburger monosyllables and multisyllables at 65 dB under free field condition) were performed around 3 months after CI activation.

Datasets were grouped according to electrode array types (CMD, FORM 24, FLEX24, FLEX28, FLEXSOFT, STANDARD). Perimodiolar CMD electrode arrays were designed for patients with intra-cochlear, intra-vestibulocochlear and trans-modiolar schwannoma. Further details about this CMD electrode, the surgical procedure and audiological details are described by Plontke et al. [21]. There were no special age and sex restrictions. For analysis all data sets were anonymized. From the four tumour patients written informed consent was received. A safety assessment was given by the responsible institutional ethical review board (approval number: 2020–149).

### Measurements

Built-in impedance tests (IFT—Impedance and Field telemetry) and its measured voltage matrices using MAESTRO 9 software (MED-EL, Innsbruck, Austria) were analysed. For IFT a defined bi-phasic pulse with an amplitude of 302.4 cu and a phase duration of 24.2 μs was applied by all individual electrode contacts respectively and the resulting voltage amplitudes between all electrode pairs were measured, individually. Each electrode contact contains an independent current source, thus using Ohm’s law the impedance is calculated in the software. The investigated data are the measured voltage amplitudes.

### Data processing

All voltage matrices were exported from the MAESTRO 9 software (MED-EL, Innsbruck, Austria) as XML file, processed and analysed with a python 3 software script.

Datasets for all CI patients were clustered depending on used electrode array types. The individual plots were visually scanned for abnormalities, like deactivated electrode contacts or shortcuts. Patients with abnormal voltage matrices, based on histogram values, were excluded from further analysis. Patients with a high number (number in histogram > 22) of data points with very low voltage amplitudes (~0 V) are excluded. For each electrode array group, the mean voltage matrix was plotted. Unpaired *t*-tests are calculated for the electrode comparisons with the python implemented scipy.stats.ttest_ind function and results are analysed with Bonferroni corrections. In addition, the line plots for the averaged CMD and the averaged FLEX28 group were plotted and discussed.

For each electrode array group, the spread of the electric field in basal and apical regions was investigated. The voltage at two neighbouring electrode contacts was plotted at electrode number 3 for apical investigation and electrode number 9 for basal comparison. *T*-tests are calculated for corresponding electrode contacts.

To analyse the correlation of monosyllable word recognition score (WRS) and electric field the user’s voltage matrices of all regular CI electrode arrays were grouped in good (WRS > 60%), medium (20% ≤ WRS ≤ 60%) and bad performers (WRS < 20%), and the voltage matrices were averaged. Unpaired *t*-tests are calculated for all three combinations with the python implemented scipy.stats.ttest_ind function.

For the CMD group, all four individual voltage matrices were shown, described, and discussed with respect to the individual speech perception improvement over the first year.

## Results

The data base contained 168 patients (29 bilaterally implanted) with datasets. Patients who got their implant in another clinic as well as children who were too young for valid monosyllable tests after three months were excluded from analysis. A total of 134 patients (4 bilateral) and the four patients with custom-made devices were analysed. The age of the patients was in average 61.2 +/- 15.6 years (median 64). Eighty patients were female. All patients are post-lingually deafened. There were four patients with the CMD FORM19 electrode array, two with FORM24, nine with FLEX24, five with FLEXSOFT, 19 with Standard and 99 with the FLEX28 electrode array. The four CMD device users had the tumour at different positions and that’s why underwent different surgeries. The locations are the following: ID1 transmodiolar, intra-vestibulo-cochlear (basal turn), ID2: intra-cochlear (middle turn and apex), ID3: intra-cochlear (basal and middle turn), ID4: intra-vestibulo-cochlear (basal, middle and apical turn). In this patient with ID1, the modular and fundal tumour parts were not removed in order to enable hearing rehabilitation with CI. For all patients the voltage matrix with the date of the three months testing (after implantation) was used.

**Fig 1:**
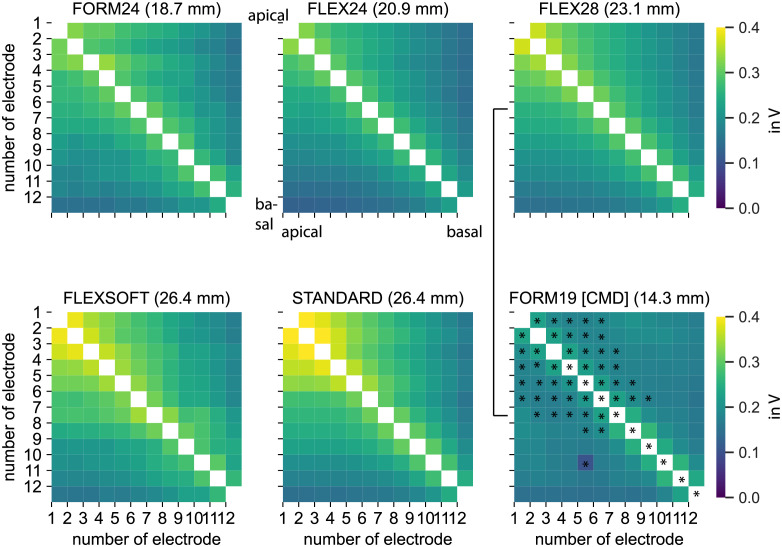
Mean voltage matrices grouped for each electrode array type (FORM24 n = 2, FLEX24 n = 9, FLEX28 n = 99, FLEXSOFT n = 6, STANDARD n = 19). T-tests show significant differences in the apical regions. Exemplary the difference between FLEX28 electrode array and FORM19 device are shown. * indicate p<0.05.

Fig 1 shows the voltage matrices grouped for each specific electrode array type. A strong voltage decrease next to the principal diagonal was observed. Compared to the perimodiolar CMD electrode arrays, larger voltage amplitudes and wider spreads of the electric field were found in all other electrode arrays. Besides, the wider electric field was more prominent in the apical region compared to basal regions (see also Fig 3). The *p-*values are calculated for all electrode combinations. Exemplary the difference between FLEX28 and CMD devices is marked with stars (*p* < 0.05). The active stimulation range differed between 14.3 mm and 26.4 mm and lead to different stimulating sites in the cochlea.

**Fig 2:**
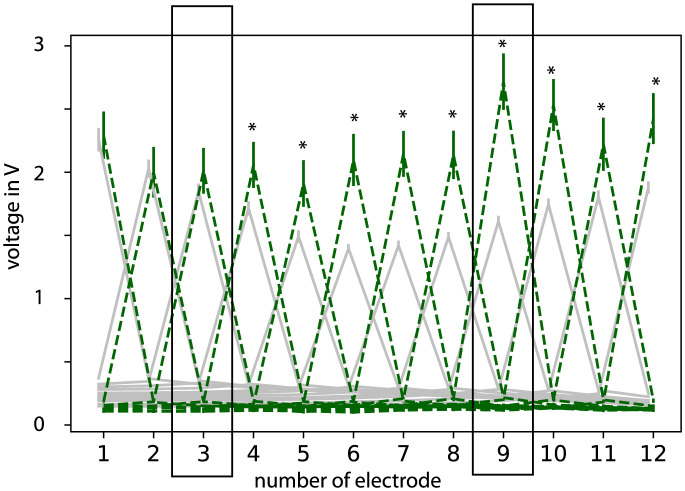
Voltage for each stimulation pair of electrode contacts. Perimodiolar CMD devices (green) and as example FLEX28 electrode averaged (grey). Error bars indicate the standard errors. The peaks are the values along the matrix diagonal. * indicates p-value < 0.05. Electrodes 3 and 10 are in rectangles because they are used for comparison of apical and basal regions in Fig 3.

**Fig 3:**
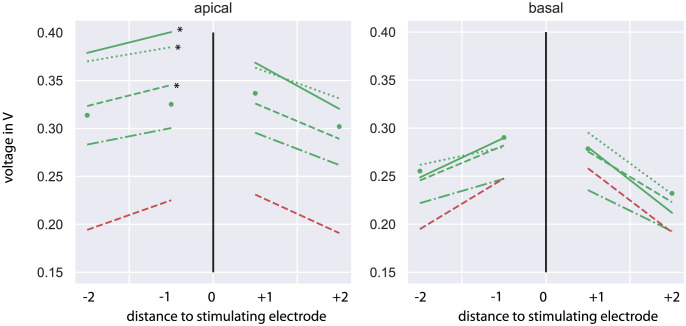
Voltages between the stimulating and adjacent electrode contacts as average across apical (left) and basal (right) stimulating electrode contacts (green: Common electrode arrays, single dots: FORM24, line-dot: FLEX24, dashed line: FLEX28, dotted: FLEXSOFT, solid line: STANDARD, red: CMD). * indicated statistical difference of electrode type compared to CMD; all other combinations are not significant p>0.05.

Fig 2 shows the voltage decrease for each electrode contact for the FLEX28 electrode array type (grey) as well as for the perimodiolar CMD arrays (green). CMD arrays showed higher and more homogeneous voltage amplitudes in the stimulation electrode contact (i.e. along the diagonal of the matrix or along the peaks in Fig 2) over the whole array compared to the FLEX28 electrode array. The voltage decrease to the neighboring electrode contact of the stimulating contact in the CMD device was higher compared to the FLEX28 electrode array. That means the voltage in the electrode contacts close to the stimulating electrode was lower for the CMD device compared to the average of other electrode arrays, especially in the apical regions (e.g., electrode contact number 2 to 1 and 3). As explanation: you see a larger difference in the diagonal (stimulating electrode = recording electrode) for electrode 4 to 12 between FLEX28 and CMD. The difference between bothe electrode types for electrode 1 to 3 is larger in the non stimulating regions. The two rectangles show the electrode areas that are pictured in Fig 3.

A closer insight in the comparison of apical and basal regions is given in Fig 3. The two neighbouring electrodes of electrode 3 and 9 are plotted. For basal electrodes contacts the voltage difference between CMD arrays and all other arrays were in the range of 0.06 V. Statistic analysis shows that there is no difference between the voltage at electrode contact 7 and 8 for CMD arrays compared with all other arrays (*p* > 0.05). The apical voltage amplitudes differed by about 0.2 V. FLEXSOFT, FLEX28 and STANDARD electrode arrays give significant different results at electrode contact 2 compared to the same on the CMD electrode array (*p* < 0.01) in the apical region, whereas between voltage at electrode contact 2 of the CMD electrode array and FLEX24 as well as FORM24 no difference is seen (*p > 0*.*05*).

**Fig 4:**
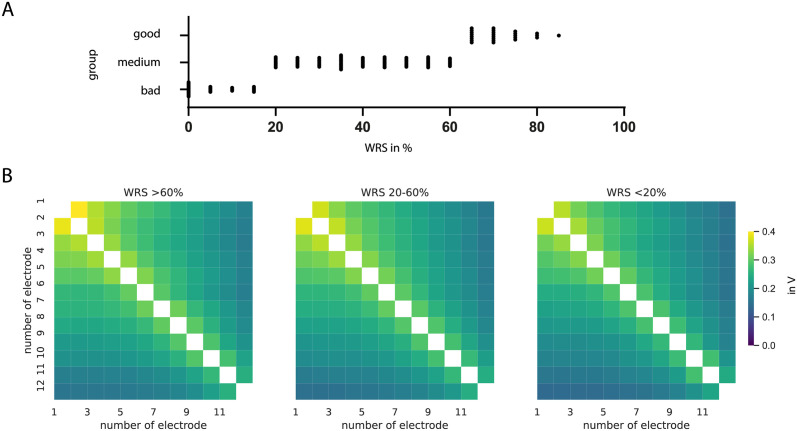
A Distribution of speech scores for all analyzed datasets. B Mean voltage matrices for different monosyllable word recognition score groups of CI users after 3 months, averaged over all common electrode array types. Monosyllable WRS better than 60% (n = 25), 20–60% (n = 78) and less than 20% (n = 31). No statistical difference was found (ps >0.05).

Fig 4 shows the mean voltage matrices for different monosyllable word recognition score groups of CI users as average over all electrode array types. The distribution of speech perception results is shown in Fig 4A. There are 25 datasets with monosyllable WRS better than 60%, 78 with WRS between 20 and 60% and 32 with less than 20%. The dominating influence comes from electrode array FLEX28 because of the number of patients. Visually, the voltage spread appears to be directly comparable between the performance groups. *T*-tests confirm this result that there is no statistical difference between the three speech perception groups.

**Fig 5:**
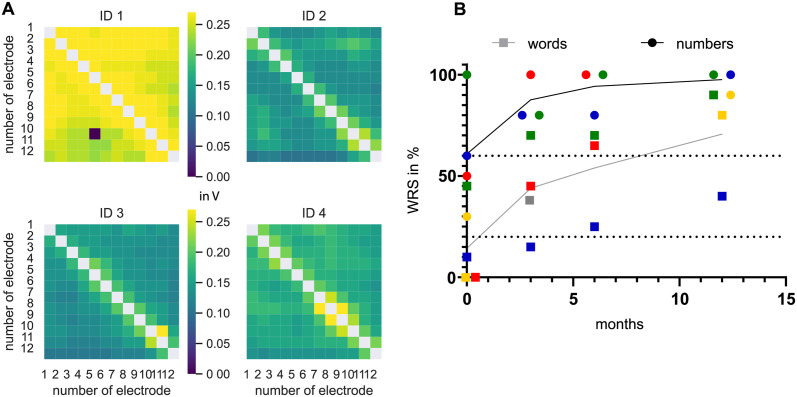
A Matrices for four patients with a perimodiolar CMD for use after surgical removal of intra-labyrinthine schwannomas (ILS). Measures after three months are shown. For detailed pictures of electrode array position see Plontke et al. 2020 [21]. B Word recognition score of CMD users. Dotted lines show the 20% and 60% monosyllable WRS line as separation between good and bad performers. ID1: green, ID2: yellow (not German speaking), ID3: red, ID4: blue. The grey line shows the averaged monosyllabic word and the black averaged multisyllabic number score for the CMD. The grey square is the averaged WRS for all users of common electrode array types.

Fig 5A shows the electric field spread for patients with a perimodiolar CMD CI electrode array after intra-cochlear schwannoma removal. A rapid decrease in voltage amplitude around the stimulating electrode contact and a rather homogenous spread over the whole cochlea was seen. The overall voltage spread differed about 0.1 V between patients ID1 and ID3. Mirroring at the diagonal asymmetries in the individual matrix were seen in all four CMD devices. Patients with a CMD electrode array showed an increase in monosyllabic word and multisyllabic number recognition scores in the first months of CI use and mostly fit to the “good” performance group (see Fig 5B).

## Discussion

The measured averaged voltage matrices for all common electrode arrays showed differences between basal and apical stimulating electrode contacts. A larger voltage gradient was observed for basal electrode contacts and is likely caused by a smaller electric spread in that cochlear region as predicted by several models describing the voltage in the cochlea [15]. In cases of regular anatomy of the cochlea these models assume a preferred current pathway through basal bone passages like the internal auditory canal, the vestibular system, the cochlear and vestibular aqueduct, and the oval as well as round window. Those models would probably not be applicable if the bone of the cochlear is removed as in the described patients after removal of an intra-cochlear tumour. After surgery, there is less fluid around and cartilage chips at the periphery of the perimodiolar electrode array, very likely fibrosis. So there is a different density and the current pathway is in apical regions as unopposed as in basal regions. Consequently, this changes electric characteristics. The CMD is pre-curved, thus it is closer to the modiolus than lateral wall electrode arrays, which means closer to the spiral ganglion cells in Rosenthal’s canal than to the bone of the remnants of the cochlear capsule, which implies a smaller resistance and a higher voltage gradient. Another effect of the pre-curved structure of the array is a deeper insertion angle which is not further investigated. This is in accordance with the findings of different voltage spreads for the CMD electrode array compared to all others. A larger decrease in voltage at the stimulating electrode contact (Fig 2) and, thus, a faster decrease in voltage in the surrounding electrode contacts (Fig 3) can be explained by the unusual surrounding of the electrode, as well as the difference in basal and apical profile.

Beside the altered electrical characteristics of the surroundings after subtotal cochleoectomy, also the electrode array structure should be considered. The apical part of FLEX electrode arrays contains a FLEX-Tip with five single electrode contacts while STANDARD and FORM electrode arrays are made of paired contacts. Therefore, differences in electrical spreads could be expected for apical electrode contacts. We observed the smallest gradient and the highest voltages for the STANDARD and FLEXSOFT electrode array whereas the FLEX28 with almost the same length showed quite smaller voltage amplitudes in the adjacent electrode contacts (see Fig 3). Nevertheless these three electrodes are the longest and they show the significant difference in the apical regions compared to the CMD array. The insertion angle for peri-modiolar electrodes in that case is with about one turn smaller than for the three longer electrode arrays with about 1.5 turns. This leads to the conclusion that the insertion depth also plays an important role for the gradient of the electric field.

In the comparison of apical spreads along different electrode arrays the data come from different stimulated regions of the cochlea. One have to consider that during interpretation of the results. The found difference between FLEXSOFT, FLEX28 and STANDARD compared to the CMD electrode array might come from the difference of the stimulated region as well as from the different distance of the modiolus.

For CMD CI users, asymmetries regarding to the diagonal were observed in the voltage matrices. These were somehow unexpected and cannot be explained in detail so far. One could suspect a difference of the measured values because of independent current sources within the electrode array and different conductivities depending on electrode surroundings and current direction. The explanation for the dark blue spot in the matrix of ID 1 (see Fig 5A) is a decoding error. This was investigated by the manufacturer. It might be due to a bad coupling during the measurement and cannot be erased afterwards. What stays unexplained is the difference in overall voltage between ID 1 and the three other CMD voltage matrices. It might be the location of the tumour in the modiolus that leads to a more radical surgery and influences the voltage spread.

### Speech perception

The electrical field spread as measured by the voltage matrices was not different among the included good and bad CI performers in this analysis. Fig 4 shows almost equal field distributions for CI users with good and poor speech perception and *t*-tests revealed no difference. An effect of voltage gradients on speech perception was not found in this cohort. One reason might be the only small variation in electrode array structures. A comparison with a larger cohort of perimodiolar electrodes might give more insight in this topic. Further one has to take into account that there are a lot of different factors influencing the speech perception and that the electric field alone is not a proven predictor for neural excitation, yet.

The few users with perimodiolar CMD electrode array showed a slightly better speech perception than the average of all other CI users (Fig 5B). This difference might be accident but could also be based on the close perimodiolar position of the pre-curved electrode array, what is one of the most significant differences compared to lateral wall electrode arrays. Also the surrounding characteristics might influence voltage spread. After subtotal cochleoectomy, the surrounding electrochemical conditions would rather be resistive than conductive. The role of both factors could not be separately analysed in this study but could be investigated in further studies comparing different electrode array shapes.

The observed speech perception in the small group of CMD CI users agrees with the usually observed growth within the first months after CI use if audioverbal therapy is applied. In this cohort all patients got the same audioverbal training after surgery.

Nevertheless it is worth to discuss the good performance of CMD ID 1. This patient has an outstanding voltage matrix after a very invasive surgery but speech perception after the first fitting is with 60% monosyllable word understanding extraordinary good. We have to emphasize that it is double checked and reliable. This extreme results are often seen after cochleoectomy and further investigation is needed to find the reasons. In contrast stay the results of ID4 below these of good performers. A very long surgery and further health limitations might be the cause.

## Conclusion

In this study, voltage matrices in CI users with different surgical techniques and electrode array types were analysed. A difference between perimodiolar (precurved, “modiolus hugging”) electrode arrays in patients after subtotal cochleoectomy and electrode arrays close to the lateral wall was found. Electrode arrays closer to the modiolus showed smaller field spreads and therefore have the potential to achieve a more focused electrical stimulation of the neurons. Not just the distance to the modiolus but also the insertion depth might have an influence. A difference in the gradient of the electric field between basal and apical regions could be shown. No effect of electric field spread on speech perception was seen in this patient cohort. Further investigations comparing different electrode array types will bring more insights in this topic.
